# Enhancing Buruli ulcer control in Ghana through social interventions: a case study from the Obom sub-district

**DOI:** 10.1186/1471-2458-13-59

**Published:** 2013-01-22

**Authors:** Collins K Ahorlu, Eric Koka, Dorothy Yeboah-Manu, Isaac Lamptey, Edwin Ampadu

**Affiliations:** 1 Noguchi Memorial Institute for Medical Research, University of Ghana, Box LG581, Legon, Ghana; 2 School of Public Health University of Ghana, Legon, Ghana; 3 Obom Health Centre, Ghana Health Service, Ga South Municipality, Obom, Ghana; 4 National Buruli ulcer control programme, Ghana Health Swervice, Accra, Ghana

**Keywords:** Buruli ulcer, Social intervention, Early case detection, Transportation, Breakfast, Social support and antibiotic treatment

## Abstract

**Background:**

Buruli ulcer is considered a re-emerging disease in West Africa where it has suffered neglect over the years, though children below the age of 16 years are the worst affected in most endemic regions. Due to delayed health seeking, the disease leads to disabilities resulting from amputation and loss of vital organs like the eye leading to school dropout and other social and economic consequences for the affected family. Early treatment with antibiotics is effective; however, this involves daily oral and intramuscular injection at distant health facilities for 56 days making it a challenge among poor rural folks living on daily subsistence work. The mode of transmission of Buruli ulcer is not known and there is no effective preventive vaccine for Buruli ulcer. Thus the only effective control tool is early case detection and treatment to reduce morbidity and associated disabilities that occurs as a result of late treatment. It is therefore essential to implement interventions that remove impediments that limit early case detection; access to early effective treatment and this paper reports one such effort where the feasibility of social interventions to enhance Buruli ulcer control was assessed.

**Methods:**

This was a qualitative study using in-depth interviews to generate information to ascertain the benefit or otherwise of the intervention implemented. Clinical records of patients to generate data to determine the feasibility and effectiveness of social interventions in the fight against Buruli ulcer was examined. In all, 56 in-depth interviews (28 at baseline and 28 at evaluation) were conducted for this report.

**Results:**

At full implementation, treatment default and dropout reduced significantly from 58.8% and 52.9% at baseline to 1.5% and 1.5% respectively. The number of early case detection went up significantly. Affected families were happy with social interventions such as provision of transportation and breakfast to patients on daily basis. Families were happy with the outpatient services provided under the intervention where no patient was admitted into the hospital.

**Conclusion:**

The study showed that with a little more investment in early case detection, diagnosis and treatment, coupled with free transportation and breakfast for patients, most of the cases could be treated effectively with the available antibiotics to avoid disability and complications from the disease.

## Background

Buruli ulcer (BU) was identified about a century ago, and it is generally referred to as a re-emerging disease particularly in West Africa, including Ghana where the prevalence in some villages is higher than tuberculosis [1]. The worst affected age group in this endemic region is mainly children aged 15 years and below. The disease begins typically as a painless nodule under the skin at the site of a trauma. In some geographical areas the first manifestation is a papule rather than the firm, painless nodule. If not treated early, the nodule gradually enlarges and erodes through the skin surface, leaving a well-demarcated ulcer with a necrotic slough in the base and widely undermined edges, the hallmark of the disease. Prolong delay, might lead to bone involvement, functional disabilities such as amputation of limbs and vital organs such as the eye. Yet many cases report at the formal sector for treatment late and various reasons have been attributed to this observation. Case fatality among BU suffers is low but they suffer a great deal of morbidity [2,3].

About 1000 cases are reported in Ghana yearly, giving a nationwide prevalence of 20.7/100,000, in 1998. However, the district level prevalence of the Ga south district where this study took place was 87.7/100,000 population [4,5].

BU is caused by the environmental pathogen *mycobacterium ulcerans*. The mode of transmission of this pathogen has eluded researchers up to date but as we wait for a better understanding of how Buruli ulcer transmission dynamics to aid disease control, it is essential to remove impediments that limit access to early effective treatment. Effective removal of impediments on the way of access to effective BU treatment requires community mobilization and involvement to determine what works from community’s point of view. This requires piloting of social interventions identified by community members to improve access to early detection and effective treatment. This will lead to the identification of those interventions that enable patients, households and community members at large to access early detection and effective treatment and reduce treatment default and dropout among patients. This paper reports one such intervention in the Obom sub-district of the Ga South municipality of Ghana.

A number of papers on Buruli ulcer have discussed socio-cultural aspects of the disease, including perceptions on causality, attitudes toward treatment and the economic burden of hospitalized patients [6-13]. However, most of these studies tend to focus on BU related beliefs, perceptions and practices of the affected people as well as the socio-economic cost of the disease and how these have affected early case detection/diagnosis and treatment seeking behaviour and other control activities negatively. Most of these studies recommended the need for Information, Education and Communication (IEC) intervention to encourage early case detection and treatment with the assumption that once people gain knowledge they will take the appropriate action to assess treatment early. In this direction, [14] stated that intensifying health education and surveillance will create awareness and encourage early treatment. However, it has been argued that “all over the world those who do not comply are those least able to comply” [15], social science studies therefore, need to go beyond the description of existing problem and believing that health education will improve treatment seeking and adherence for BU. The real issues are the availability of care and the ability of the affected persons to overcome the barriers to assessing effective early diagnosis and treatment.

Until 2006, BU was treated mainly by surgical intervention, necessitating centralised management of cases at bigger health facilities. However since 2006, the WHO recommended first line treatment for BU is oral rifampicin (10 mg/kg) plus intramuscular streptomycin injection (15 mg/kg), both given daily for 8 weeks under supervision. Although daily injections for eight weeks is not a pleasant experience to go through, it has proven to be very effective in curing BU patients effectively, especially when accessed at the early stage of the disease. The paper reports on the barriers to accessing this effective BU treatment regimen and interventions implemented to remove these barriers through social support, which motivate patients to access and complete treatment once initiated.

This paper reports on the effectiveness of community involvement and social interventions to facilitate early BU recognition and referral to the clinic for early diagnosis and treatment. Findings were discussed in line with access to health care framework [16]. This framework looked at access to health care in terms of availability, accessibility, affordability, adequacy and acceptability of the service that clients are expected to receive from the facility.

## Methods

### Study Area

The study was conducted in the Obom sub-district of the Ga South municipality. The Municipality has an estimated population of 210,727 living in 362 communities. The municipality has a number of peri-urban settlements due to its proximity to Accra, the national capital. Ga South municipality is highly endemic of BU and together with Ga West municipality (both municipalities were formerly together and was known as the Ga district until 2009 when it was split into two as we have them today) continue to report the largest proportion of severe form of ulcerative BU cases in Ghana. The Obom health centre is the second largest health facility in the Ga South Municipality beside the Weija hospital. There are few family planning and private clinics as well as maternity homes in the municipality.

### Study design

This was a qualitative study designed to determine the effectiveness of community involvement and social interventions to enhance early diagnosis and treatment by comparing baseline and evaluation findings. However, to put the qualitative narratives in perspective, facility records were reviewed to generate data on hospitalization at the only hospital with BU wards in the Greater Accra region. Two sets of related interventions were implemented and evaluated at different times. The first intervention phase was from July to December 2010 and comprises; community outreach education and screening, re-training of community-based volunteers who were given bicycles, the provision of transportation to convey patients to and from clinic for daily treatment. The second intervention was from January to June 2011 and comprises all the interventions implemented at the first phase in addition to; formation of former BU patients’ club; training and collaboration with traditional healers and provision of breakfast to patients after treatment at the clinic. Baseline period was from January to June 2010. This period was chosen because there was very little (incomplete or no) record on the few patients seen at the clinic, hence the decision to start from January 2010, where records were available on patients.

The aim of all these interventions was to enhance early case detection, diagnosis and treatment at the Obom health centre to reduce treatment dropout and default.

### Interventions

All the various interventions were implemented with the aim of improving early case detection/diagnosis, treatment/management and ensure treatment completion through enhanced community involvement and support by increasing access to health care services for BU patients. Interventions also aimed at supporting the health care system (clinics) to respond promptly and appropriately through early laboratory testing and treatment. The implementation period was divided into two for two reasons. First, to make the evaluation periods comparable to the baseline (six months) and secondly, to take care of additional interventions introduced during the full implementation period.

1. **Community outreach to enhance early case detection and treatment**: This intervention involved showing of BU related documentary films and pictures, especially those depicting success stories of biomedical treatments during the early hours of the night usually between 7 and 9 pm. The documentary film is interspersed with questions and answers. This was followed by early morning mass screening for suspected BU cases. This was done by a team from Noguchi Memorial Institute for Medical Research supported by the environmental health officer in the sub-district and community assistants. Samples were taken from all suspected cases for laboratory confirmation in the laboratory at the Noguchi memorial Institute for medical Research.

2. **Re-training of community-based volunteers in BU case detection and referral to the clinic**: Community-based volunteers were selected and trained by the Ghana Health Service, however it was realized that they were not active in the communities. So, sixteen of them within the study area were re-trained and commissioned to be engaged in BU case searching and referral to the clinic for sampling and subsequent treatment. As a way of boosting their morale, 12 out of the 16 were given new bicycles to aid their movement within their catchment areas in search of cases. The remaining four reported having their own bicycles and asked for support to replace the tyres, which was granted. During the period of the second implementation, an amount of GH¢10 ($7) was paid to volunteers for every case referred to the clinic and confirmed to be BU. However, when a referred case is not confirmed to be BU at the laboratory then, only the transportation cost for bringing the suspected patient to the clinic was covered. This was done to reduce or avoid the system being abused for monetary gains.

3. **Formation of BU former patients clubs to promote early case detection and referral to the clinic**: In order to encourage former patients to serve as biomedical treatment ambassadors in their communities, three former patients clubs were formed in the communities to promote early case finding and referral to the clinic for early diagnosis and treatment while promoting the virtues of early BU treatment at the clinic. As in the case of the volunteers, an amount of GH¢10 ($7) was paid for every case referred to the clinic and confirmed to be BU. Membership into the club is voluntary.

4. **Provision of transportation to convey BU cases to and from the clinic**: Transportation difficulties both in terms of availability and cost were contributing to treatment default and dropout as well as affecting school attendance among those in. So transportation was provided to convey confirmed cases to and from clinic on daily basis to ensure adherence and make it possible for those in school to return to school early enough not to miss any lesson.

5. **Provision of breakfast (snacks) to BU patients after drug administration on daily basis**: During the baseline study, patients complained that they feel hungry after taking the medications but have no money for feeding themselves and this was one of the disincentives for clinic attendance. So arrangement was made to provide breakfast to patients some minutes after taking the drugs to encourage clinic attendance and adherence to treatment on daily basis. The breakfast cost GH¢1.00 ($0.70 cents) per person.

6. **Training and collaboration with traditional healers**: Ten Traditional healers were trained in BU case detection and referral to the clinic. Before the training, meetings were held with the traditional healers in the study area to discuss possible collaborations in BU case detection and referral to the clinic. This was done in a manner that ensured mutual trust and respect in order to enhance BU patients’ access to biomedical treatment, especially those who would have ended-up in the shrines of traditional healers. As in the case of the volunteers and former patients, an amount of GH¢10 ($7) was paid for every case referred to the clinic and confirmed to be BU.

### Data collection

#### Baseline data collection

1. **In-depth interview**: In-depth interviews were conducted with selected BU patients, former patients and caretakers of BU patients as well as community volunteers, traditional healers and clinic staffs to solicit information on general attitude to BU, expectations about biomedical treatments, early case detection, referrals, diagnosis, treatment and the challenges facing these processes. In all, 28 in-depth interviews were conducted (6 BU patients; 4 former patients; 6 family caretakers of BU patients; 4 traditional healers; 6 community volunteers and 2 clinical staff). With the exception of the traditional healers who were all men, women were equally represented in all the remaining groups. BU patients, former patients, family caretakers, community-based volunteers and traditional healers were selected randomly while the clinic staffs were conveniently selected to represent those who take care of BU patients in terms of drug administration, wound dressing and feeding.

In-depth interview is a flexible way of generating data as it allows for the collection of detailed information from individuals, probing important related issues as they came up concerning the topics of interest during the interview session. An open-ended in-depth interview guides were developed, pre-tested and used for data collection. The guides helped the interviewer to stay on course and managed deviations effectively. Two research assistants together with the first author (CKA) conducted the interviews. At all the sessions, one person act as a note taker and with permission, the interviews were tape-recorded.

2. **Hospital/clinic records review:** In order to be able to demonstrate changes that occur when the interventions were implemented, clinical records of patients were reviewed (January – June 2010 – baseline; July – December 2010 – first implementation and January – June 2011 – second implementation). During the reviews, treatment card of all patients treated were examined to record treatment starting date, defaulting dates, dropout dates and completion dates to determine actual treatment status of individual patients. And to better appreciate the concern raised by respondents at baseline about the effect of long hospital admission on health seeking behaviour, a review was done at the only hospital with BU wards in the greater Accra region. This hospital continues to receive patients from both far and near. The review was done to determine the length of hospital stays during admission to understand why it was a problem for the affected households.

### Evaluations

Monitoring and evaluation of the programme at facility and community levels were integral parts of the implementation. Weekly visits and monthly meetings were held with stakeholders to assess the progress of work and address emerging issues to ensure smooth implementation. The In-depth interviews were conducted before and after implementation of interventions. Thus, results from the baseline informed the choice of interventions implemented. Just like the baseline, 28 In-depth interviews were conducted at evaluation using similar interview guides. Also clinical records were reviewed at both baseline and evaluations to examine the number of patients treated or being treated. This allowed the determination of the proportions of patients that dropped out of treatment or defaulted on their treatment but managed to complete it at one point or the other.

### Data analysis

Qualitative data from in-depth interviews were entered into a word processor (Microsoft Word) and imported into MAXqda in a format that allows automatic coding by interview item. MAXqda is a programme for textual analysis. Data was analysed to clarify aspects of illness-related experience, meaning and behaviour. Variables of interest in the qualitative data-base were imported into MAXqda as selection variables. This allowed the performance of phenomenological analysis on relevant coded segments from respondents for presentation. Representative narratives were presented to show the position of respondents on topics of interest. Where appropriate, narratives were used to complement and clarify the quantitative categories for a better understanding. Quantitative analysis was done using Epi Info (version 3.3.2) software to generate descriptive statistics for presentation.

### Ethical review

The Institutional Review Board of the Noguchi Memorial Institute for Medical Research, University of Ghana reviewed the study. Each participant/caretaker was informed of the objectives, methods, anticipated benefits and potential hazards of the study. Participants/Caretakers were also informed that they were at liberty to withdraw from the study at any time without penalty. They were assured that all information collected during this study would be kept confidential and that in any resulting publication it would not be possible to link the data to individuals in the study.

Community consent was sought from chiefs and elders of all participating communities to perform case search, early recognition, diagnosis and treatment in the communities. Oral informed consent was sought from adult suspected cases and from caretakers of children under 18 years before samples were taken from them for laboratory diagnosis (the laboratory component is another arm of the study which may be reported elsewhere). Results from the laboratory was communicated to patients and caretakers after which another oral consent sought from those who were confirmed as having BU infections to be recruited into the social intervention study. Oral informed consent was preferred because most of the respondents could hardly read or write.

## Results

### Social factors affecting clinic attendance

Hospital or clinic attendance for BU treatment is often influenced by social considerations. Caring for other family members, especially in large families, where there are other younger children to be taken care of, and considering the fact that most of the affected families live on subsistence farming, spending time at the clinic means losing out on daily farm activity, this was an unpleasant thing to do. This position was reflected in a statement by a mother during baseline, when she said;

“...Sometimes you would want to go to the clinic but besides the money for transportation, you also find it difficult to leave other children at home because you are not sure that you will go on admission or you will be allowed to comeback. ...You have to go to the farm to bring home food for the family as food stuff like cassava is better harvested on daily basis to avoid spoilage…”(a mother of four, in-depth interview).

The above sentiments changed after the interventions were implemented as demonstrated in the following representative narrative, when a lady at the final evaluation said;

“…since the introduction of the free breakfast and transportation, it has become easier for us to attend clinic as we do not have to worry about breakfast or transportation cost. ...one is able to go to farm or school after clinic attendance and this is good” (mother of an infected child, in-depth interview).

Concern about marital responsibilities also prevented mothers from taking the affected child to the clinic, especially when they perceive that the child will be admitted at the hospital. Here the emphasis is not on the children left behind at home alone but also the husband who they fear may go after other ladies in their absence. This position is put forward by a lady with four children with one of them having BU when she said;

“The problem is about you men (referring to the interviewer), some of you cannot wait for your wives to travel before you start chasing other women in town and some of us cannot tolerate that because before you know it, your husband is having another wife…” (mother of infected child, In-depth interview).

However, this was largely addressed by the interventions as reported by a mother in the following narratives;

“...Now, I do not have to go with my son to the clinic, all I have to do is make sure that he bathed and dressed for school..as he will be taken by the car free of charge and will be given food at the clinic...taken to school on his way home...I can then take care of his father and siblings as well as go to the farm to work…” (mother of infected child, In-depth interview).

BU sufferers, especially the children suffer from stigmatization both in school and in the community and this were contributing to people hiding the infection just to avoid being teased, especially at school. A school girl said;

“…When I first saw the wound (BU infection), I did not want anybody to know about it, not even my parents until it became very painful…… I was afraid that my friends will be teasing me and that will make me stop going to school” (infected school boy, In-depth interview).

Attitude of health workers also came up as a factor influencing hospital attendance as represented in the following statement by a lady;

“I went to the clinic at …before, but I stopped going because they behaved as if my whole body smells foul and some of the nurses were spitting and holding their noses, I felt sad and embarrassed because it was as if I was the cause of my own problem ... But you see, this disease, it can affect anybody…I used to think that it will not affect me but here am I, with this big sore on my body…” (infected lady, In-depth interview).

At the end of the intervention, however, this is what a man said in an in-depth interview;

“...Oh, now the nurses are very nice with us, they even serve us food every morning after treatment...we are now friends... The Chief (medical assistant) is a very good person, he tells us to try our best to finish the injection as it is good for us…” (infected man, In-depth interview).

One major restriction to seeking health at the clinic/hospital was the fear of surgery, especially amputation. This position was represented in the following narratives by a lady respondent when she said;

“…I know someone who went to the hospital on his two legs but came back with only one leg …I do not want to be walking in the village with only one leg neither will I be happy to see any of my children with one leg… as for the local healers, they do not cut off patients legs…, that is why they are preferred in most cases. If the hospital will stop cutting off the legs of the patients…we shall go for treatment” (a mother of five, in-depth interview).

However at the final evaluation, a lady said;

“... the difference between here (intervention clinic, Obom) and Amansama (the district capital of the adjacent district where BU ward exist within the district hospital) is that you are not admitted into the hospital, where your leg or hand could be cut off...here they dress your wounds and give you injection, you eat and go home or school to your family and friend…this is good for us” (infected lady, in-depth interview)

Long hospital stay is another factor that keeps people away from going to the hospital as narrated by a man in an in-depth interview session when he said;

“…the doctors keep the patients at the hospital for too long… this usually affect other things, especially when the mother of the house have to stay with the patient for that long period at the hospital…, to be able to do that one has to find another person to take care of the rest of the family. As a man, I cannot go and stay at the hospital with my child…who will work to take care of the family? The presence of the mother at home is equally important…” (affected man, in-depth interview).

At the final evaluation, a lady said;

“since I started coming for treatment, nobody has been referred to Amansama for admission...all of us come to get our injections and have our wounds dressed then allowed to go home, I think that if all the hospitals/clinics are treating us like Obom, everybody will be happy to come for treatment. ..What I used to fear most was going on admission...” (Infected lady, In-depth interview)

In order to ascertain why long stay at the hospital during admission was considered a problem by the people, the hospital admission record at the Amansama BU ward was reviewed. The result revealed that, the length of hospital stays varied from about a month to more than10 years in few cases. Majority 134 (76.3%) were discharged within 12 months, however, the few who stayed beyond 12 months, especially the 29 (16.3%) who stayed for over three years should call for concern indeed, as they became the reference point for affected individuals and families (Table 1).

**Table 1:** Length of hospital stays at Amansaman Bu ward

**Duration**	**Number of Cases N=177 (%)**
Up to 1 month	41 (23.0%)
2 to 6 months	66 (37.1%)
7 to 12 months	28 (15.7%)
13 to 36 months	14 (7.9%)
37 to 72 months	16 (9.0%)
73 to 120 months	9 (5.1%)
>120 months	4 (2.2%)

### Economic factors affecting BU related health seeking behaviour

Residents in most affected communities are subsistence farmers whose daily survival depends on what they get from the farm, mostly on daily basis therefore, one main consideration for seeking health at the clinic/hospital is ‘taking time off work’ (farm, market or in the sand pit). In fact, ‘taking time off work’ usually mean less food for the day, especially during the lean season. They also claimed that at times they may have cassava in the farm but their inability to go and fetch it as a result of going to the clinic may mean that they have very little to eat when they return, that is if they were not admitted into the hospital. This position was represented by a view shared by an affected mother;

“As you can see, we bring our foodstuffs home on daily basis, so if you miss going to the farm one day, it affects how much food you have at home... Our husbands are paid on daily basis from the sand pits... So it is difficult to leave say 4 or more healthy children at home to starve when you take one to the hospital..” (affected lady, in-depth interview).

However, at the final evaluation, one man said in an in-depth interview;

“...the current arrangement is what is good for us ...I work in the sand pit ...my wife works on the farm...it was difficult taking the child to the clinic but now the driver pick him every day to and from the hospital free of charge..who will not like this…?” (affected man, in-depth interview).

Lack of access road within the sub-district also affect health seeking behaviour as it makes movement to and from the clinic very difficult hence very prohibitive transportation cost making it unaffordable to many people on daily basis. A gentleman in one of the communities said;

“ …Getting vehicle to travel to the clinic is one major problem. You see, I cannot walk on this leg to go to the hospital which is far way, about 8 km, I will die on the way, Our roads are bad and no vehicle comes here, especially during the raining season. Even if a car managed to come, we cannot afford to go daily…… they charge too much” (affected young man, in-depth interview).

During the final evaluation however, this was what a male community leader said;

“…… I think the best thing that has happened to us in this community is the provision of transportation to take BU patients to the clinic and back……we shall remain grateful and hope that you will continue to help us……to be free from this bad disease……so that our younger children will not suffer from it” (a male community leader, in-depth interview).

### Referral to the clinic

At baseline, most 10 (58.8%), of the cases treated went to the clinic either by themselves or referred by relatives. At the end of the partial intervention however, most 28 (70.0%) were referred by the research team through community outreach programmes. On the other hand, at the end of full implementation, 30 (38.0%) were referred through community outreach programmes and 25 (32.6%) by community-based volunteers (Table 2). It is worth reporting that there was a major increased in the number of patients referred by community-based volunteers from four at baseline to 25 by the end of full implementation, an increase of over 600%. Also, cases referred through health outreach activities have increased from two at baseline to 30 at the end of full implementation (Table 2).

**Table 2:** Who referred patients to the clinic?

**Variables**	**January–June 2010 (Before intervention) N = 17 (%)**	**July–December 2010 (Partial intervention) N= 40 (%)**	**January–June 2011 (Full intervention) N= 79 (%)**
Community outreach	2 (11.8)	28 (70.0)	30 (38.0)
Community volunteers	4 (23.5)	4 (10.0)	25 (31.6)
Former patient	0	1 (2.5)	7 (8.9)
School teacher	1 (5.9)	1 (2.5)	1 (1.3)
Self/relative	10 (58.8)	5 (12.5)	14 (17.7)
Traditional healer	0	1 (2.5)	2 (2.5)

Most of the cases referred during the intervention period were classified as early reporting, thus, classified by providers as category one or two, where category three is considered as late reporting. Specifically, about 70% of the cases referred through community outreach activities were classified as early reporting compared to none at baseline. Also, about 55% of cases referred by community volunteers were classified as early reporting compared to none at baseline.

### Diagnosis and treatment

At baseline (January – June 2010) 17 cases were diagnosed and treated, by the end of the partial implementation, 40 cases were diagnosed and treated (June – December 2010) and by the end of full implementation, 79 cases were diagnosed out of which 68 were treated (the remaining 11 cases did not start treatment because they moved out of the study area before lab confirmation was completed). Further investigations revealed that they came to spend holidays with their relatives (Fisher-folks) living along the Weija Dam.

Clinic records at baseline showed that between January and June, 2010, 10 out of the 17 cases treated were classified as early diagnoses while at the end of the partial implementation between July and December 2010, 22 out of the 40 cases treated were classified as early diagnoses. However, at full implementation period between January and June 2011, 62 out of the 79 cases were classified as early diagnoses, but 11 of them did not get into treatment leaving 51 out of 68 being treated as early stage cases. The number of cases that were treated early increased from 10 at baseline to 51 at the end of full implementation. An increased of about 500% in one year. This was well represented in the following statement “*As people who take care of these patients when they come, especially dressing their wounds, I can say that the current intervention is very good..Now, more people are coming with small wounds which are very easy to manage and we are all happy* (clinic staff, In-depth interview).

### Treatment default

At baseline, 10 (58.8%) out of the 17 patients defaulted on treatment at one point or the other, while during partial intervention, 15 (37.5%) out of 40 defaulted on treatment schedules whiles only 1 (1.5%) out of the 68 patients who started treatment has defaulted and a follow-up investigation revealed that he moved out of the study area completely.

### Treatment dropout

At baseline, nine out of the 17 patients did not complete treatment. An indication that one of those who defaulted on their treatment schedules also managed to complete treatment. By the end of partial implementation 10 (28.7%) out of the 40 patients dropped out of treatment, thus five out of the 15 defaulters managed to complete treatment successfully. However, only 1 (1.5%) out of 68 patients had dropped out of treatment by the end of full pilot implementation. This gave the proportion of those who successfully completed treatment as eight out of 17 (baseline), 30 out of 40 (partial implementation) and 67 out of 68 (full pilot implementation).

## Discussion

This paper moved away from the most frequently reported social science studies on BU with emphasis on socio-cultural aspects of the disease including perceptions, attitudes toward treatment and economic burden of disease to patients and their families [6-8,10,12,13] to focus on how to increase access to early case detection, diagnosis and treatment as well as how to reduce treatment default and dropout during the course of 56 days antibiotic treatment. Building on the BUPaT project [14]; the social intervention was to remove socio-economic challenges to accessing treatment once diagnosis was made. However, this project was different from BUPaT in many ways as treatment/management was organized on an outpatient basis with no hospital admission; also daily breakfast and transportation were provided for each patient. Results reported in this paper showed that when various interventions are implemented together, they could enhance effective early case detection; diagnosis and treatment with little default and drop out. Whereas, the BUPat project has reported challenges with lack of hospital ward space [14], this was not the case in the current intervention as patients were supported to come from home to the clinic on daily basis. While in the BUPat project caretakers accompanied patients to the hospital and stay with them as long as the patient remained on admission, thus raising the issue of space at the wards for patients and accommodation for caretakers [14]. This also took caretakers away from their daily subsistence work. The current study did not subject caretakers to such activities nor were they taken away from their daily works, thus caretakers were not on ‘admission’ together with the patients and therefore able to take care of other pressing needs while the patient receive treatment at the hospital on an outpatient basis.

The provision of breakfast and transportation means that school children could receive daily treatment on time, have breakfast and be conveyed back to school on time in order not to miss any lesson and thereby removing the incentive of dropping out of school.

The relevance of the five dimensions of access to health care [16] cannot be overemphasized. Availability issues were addressed in this intervention by ensuring that the intervention health facility at Obom was ready to deal with the health needs of BU patients when referred there. To achieve this, training was organized for the staff on BU management including sample taking and wound dressing. Also, some basic supplies were made available to the health centre from time to time to ensure that they do not run out of stock necessary to meet the needs of BU patients effectively. In terms of accessibility, the project ensures that BU management services are provided at a location acceptable to patients. However, the challenge of accessibility was addressed by the provision of free transport to carry patients to and from the clinic on daily basis. Affordability, which deals with the ability of patients to pay for the cost of the health care service being provided with very little or no difficulty, was removed completely because BU treatment is generally free in Ghana and all other associated cost including transportation were taken care of by the Stop-Buruli project. The issue of adequacy was addressed by making sure that whatever services that are being provided meet the needs and expectations of BU patients. To make the service acceptable, providers were encouraged and motivated to develop attitudes and atmospheres that make BU patients feel at home and willing to come to the clinic on daily basis for 56 days or more.

The number of cases referred by community-based volunteers had increased significantly during the implementation of the intervention. This increase could be attributed to the incentive schemes put in place to reward hardworking volunteers. Besides the token given for each case referred, the provision of bicycles to them for easy movement within the community had also contributed to the surge in cases being referred. This has confirmed the fact that for volunteers to work effectively, a reward system is required to reward hardworking ones among them. These incentives do not have to be anything too big but the mere fact of knowing that one’s work is being recognized and appreciated is enough incentive to most community based volunteers.

Traditional healers did not contribute much to case referrals but it was symbolic to collaborate with them as this could influence what they do and how long they may keep patients before referring them to the clinic. In the light of this, the three cases referred by them should be seen symbolically as a good starting point taking into account the fact that they were not referring any case prior to the implementation of the intervention.

It has been demonstrated from this study that it was not only possible to increase BU early case detection and diagnosis but also to effectively treat BU patients on outpatient basis at health centres to significantly reduce in-patient pressure on the health facilities in terms of space for admission. Hospital admission do not only put pressure on the health facility but also on the family caretakers who have to stay on ‘admission’ with the patients for as long as it takes to get the patient discharge from the hospital [14]. In this study, caretakers were not even accompanying patients to the health centre and this made it possible for them to go about their normal daily works while patients receive effective treatment from the health centre.

For programme implementers, the need for retaining community based volunteers, the formation of former patients clubs, organization of community outreaches education and screening are recommended to enhance early case detection and treatments. However, in order to encourage patients from poor communities to start and complete treatments, the provision of transportation to and from clinic as well as breakfast after drug administration could enhance attendance to reduce treatment dropout and default significantly.

In implementing interventions such as reported here involves some cost but the cost of this intervention should not be over exaggerated because in the long run it may be cheaper to implement these activities to diagnose and treat patients early as outpatient than to receive them late and keep them on admission, which may require surgical interventions in some cases. Given that only about 2000 cases are reported annually in Ghana and assuming that this number will go up to about 2500 annually due to improved active case searching, it should be possible for the Government, through the district assemblies and in collaboration with the Ghana health service, to raise sufficient funds to support this important intervention in poor communities. Early case detection will prevent surgery in the future and this will save a lot of money for the health system as most of the cases will get healed with antibiotic treatment and adequate wound care”. BU affects mostly the poorest of the poor and this should be enough motivation for implementing interventions to reduce the disease burden on this vulnerable population. However, there is a need to do cost effectiveness analysis to determine the cost benefits of the interventions reported in this study.

For interventions such as reported in this paper to be successfully implemented, there is a need to involve stakeholders, especially community groups and health care workers at the periphery facilities who will attend to the patients when referred. Involving the local people will help us to know their needs instead of assuming that money is their problem. In fact, doing so, we realized that the patients do not need money but a means of transport and readily available breakfast to take after drug administration, hence the introduction of these two social interventions. Health facilities could easily be overwhelmed by the number of cases that they see on daily basis and if they are not involved in planning for these cases, could affect the quality of care that are given and this may work against the intended purpose of the intervention.

### Weaknesses of the study

One major weakness of this study is that it was not a randomized control trial and therefore one could not tell the actual contribution of each intervention to the increased number of cases seen at the clinic as well as the near elimination of treatment default and dropout. Also, the study was purely qualitative besides the clinic records evaluated therefore results could not be generalized. However, we believe that this has been compensated for by the baseline data and the presentation of qualitative narratives to express the actual feelings of respondents.

## Conclusion

In conclusion, our results suggest a role for breakfast and transport cost reimbursement to improve compliance. It was clear that the provision of breakfast and free transport to convey patients to and from clinic has contributed significantly to the near elimination of treatment default and drop out among patients in our study. It is therefore worth investing about 85 dollars (this amount include; motivation for the one who referred the case, transportation and breakfast for a minimum of 56 days) on each patient, especially to support the poorest of the poor, in remote villages where BU is mostly endemic to receive and complete treatment. These support also helped school children to continue with their education while receiving effective treatment for a debilitating condition like Buruli ulcer.

## Abbreviations

BU: Buruli ulcer; WHO: World Health Organization; IEC: Information education and communication; UBS: United Bank of Switzerland.

## Competing interests

The authors declare that they have no competing interest.

## Authors’ contributions

CKA was involved in the conception, design and implementation, data analysis and writing of this paper. EK was involved in the implementation, data collection and writing of this paper. DY-M was involved in the conception, implementation and writing of this paper. IL was involved in the implementation and data collection. EA was involved in design and implementation. All the authors have read and approved the final version of the manuscript.

## Pre-publication history

The pre-publication history for this paper can be accessed here:

http://www.biomedcentral.com/1471-2458/13/59/prepub
